# Ten-year outcome of Eculizumab in kidney transplant recipients with atypical hemolytic uremic syndrome– a single center experience

**DOI:** 10.1186/s12882-020-01847-0

**Published:** 2020-05-20

**Authors:** Sam Kant, Anshul Bhalla, Sami Alasfar, Nada Alachkar

**Affiliations:** 1 Department of Medicine, Division of Nephrology, The Johns Hopkins University School of Medicine, 600 N Wolfe St, Carnegie 344B, Baltimore, Maryland 21287 USA; 2 Department of Surgery, Division of Transplant Surgery, James D. Eason Transplant Institute, Methodist University Hospital, University of Tennessee Health Science Center, Memphis, TN USA

**Keywords:** Eculizumab, Atypical hemolytic uremic syndrome, Kidney transplant

## Abstract

**Background:**

Atypical hemolytic uremic syndrome (aHUS) can result in severe kidney dysfunction, secondary to thrombotic microangiopathy. Eculizumab has been used to treat this disorder, and has resulted in favourable outcomes in both, native and transplanted kidneys. There is limited long term follow up data in kidney transplant recipients (KTRs) who received prevention and treatment with Eculizumab. We report our long term follow up data from our center to address safety and efficacy of this therapy in KTRs.

**Methods:**

We performed a retrospective analysis of KTRs between January 2009 and December 2018. Clinical diagnosis of aHUS established with presence of thrombotic microangiopathy, acute kidney injury, absence of alternate identifiable etiology. We reviewed clinical data, including genetic testing for complement factor mutations, post-transplant course, and response to therapy including therapeutic and prophylactic use of eculizumab.

**Results:**

Nineteen patients with aHUS received a total of 36 kidney transplants; 10 of them had 2 or more prior kidney transplants. Median age at time of last transplant was 37 years (range 27–59), 72% were female (*n* = 14), 78% Caucasian (*n* = 15), with 61% had live donor transplant (*n* = 12) as the last transplant. Eculizumab prophylaxis was given to 10/19 (56%) at the time of transplantation, with no aHUS recurrence during the follow up. Median duration of follow up was 46 (range 6–237) months. Mean estimated glomerular filtration rate (eGFR) at the time of last follow up was 59.5 ml/min/m^2^. No infections secondary to encapsulated organisms or other major infectious complications occurred during the follow up.

**Conclusions:**

Eculizumab prophylaxis is safe and effective in KTRs with aHUS. Long term follow up demonstrates that it may be possible to discontinue prophylaxis carefully in selected patients with no evidence of complement mutations.

## Background

Atypical hemolytic uremic syndrome (aHUS) is a rare disorder characterized by thrombotic microangiopathy, as a result of abnormal activation of alternate complement pathway [1]. Ensuing endothelial injury can lead to severe kidney dysfunction, with a propensity for progression to end stage renal disease (ESRD) and recurrence of the disease after kidney transplant [2], Complement inhibition using eculizumab, a humanized C5 monoclonal antibody, has been demonstrated to aid in recovery of kidney function in aHUS in native and transplanted kidneys, along with preventing recurrence [3–10].

Recurrence of aHUS continues to be a major issue in the post-transplant period and even many years post transplant. Recovery of renal function as well as normalization of hematological parameters was lower for aHUS episodes involving transplanted than native kidneys [4, 11]. Risk factors include genetic complement mutations (in particular, complement factor H) and previous history of recurrence in kidney transplant recipients (KTRs) [12]. Other factors like ischemia reperfusion injury, immunosuppressive medications and infections can also trigger aHUS recurrence. Commencement of prophylactic therapy with Eculizumab has been advocated in patients at moderate to high risk of recurrence, however, timing of treatment with relation to transplantation or duration of treatment has not been established [2]. Long term data regarding the safety and the efficacy of eculizumab therapy in this patient population is also sparse.

We aim to report our 10-year experience of management of aHUS in KTRs, utilizing Eculizumab in the prevention and the treatment of the disease recurrence.

## Methods

### Study population

We included all adult (age ≥ 18 years at time of their last kidney transplant) KTRs with aHUS who were transplanted at Johns Hopkins Hospital from 2009 to 2018. This study was approved by the Johns Hopkins Hospital Institutional Review Board (IRB). Clinical diagnosis of aHUS established with presence of thrombotic microangiopathy, acute kidney injury, absence of alternate identifiable etiology. We reviewed clinical data, including genetic testing for complement factor mutations, post-transplant course, and response to therapy including therapeutic and prophylactic use of eculizumab and long-term allograft outcome.

### Induction and maintenance immunosuppression

Standard induction therapy was anti-thymocyte globulin (Thymoglobulin, Genzyme) 1.5 mg/kg/day for 5 days and high-dose steroids. The typical steroid regimen was methylprednisolone 500 mg intraoperative, followed by a 50% taper daily for three days. Recipients were then switched to oral prednisone 30 mg/day by post-operative day five. Recipients felt to be at high-risk (e.g. presence of DSA or repeat mismatches) were also given a single dose of anti-CD20 antibody (Rituximab, 375 mg/m2) at time of transplant. Standard maintenance immunosuppression was tacrolimus (goal serum level of 8 to 12 ng/mL in the peri-operative time period), mycophenolate mofetil (2 g/day), and prednisone (tapered to 5 mg/day by three months post-transplant).

### aHUS diagnosis and treatment

The diagnosis of aHUS recurrence or de novo post transplant was confirmed by kidney transplant biopsy confirming thrombotic microangiopathy (TMA). Eculizumab prophylaxis protocol at time of transplant: 1200 mg peri-operative initial dose, subsequently 900 mg weekly for four weeks, then 1200 mg on week 5, to continue 1200 mg every 2 weeks. Treatment of recurrent and de novo aHUS post transplant included plasma exchanges followed by intravascular immunoglobulin (IVIg) 100 mg/kg after each plasma exchange session. Additionally, eculzimuab was started at 900 mg weekly for four weekly followed by 1200 mg on week five to continue 1200 mg every two weeks. In those who required plasma exchnage, each session was also followed by eculizumab 600 mg. The duration of plasma exchnage was dictated by the treatment response relying on the hematological parameter, renal function and in some cases pathology. Patients received between 5 and 10 plasma exchange sessions.

All the patients who received prophylactic or thereapeutic eculizumab also received meningococcal vaccine. Those who received eculuzimab for five years or more, were vaccinated again with a meningococcal vaccine boost. In addition to the vaccine, we elected to have the all the patients on prophylactic antibiotic with amoxicillin (or equivalent in cases of penicillin allergy).

### Outcomes

We studied mortality and death-censored graft loss, which censors recipients who died with a functioning graft. We determined cause of death, or graft loss, through medical chart review. We also characterized long-term graft function through longitudinal serum creatinine measurement. We also assessed the side effects of long term use of eculizumab.

### Statistical analysis

The cumulative incidence of mortality and death-censored graft failure was determined through Kaplan-Meier analysis. Date is presented in years. All analyses were performed using Stata 15.0/IC for Mac (College Station, Texas).

## Results

We identified 19 patients with aHUS who received a total of 36 kidney transplants (Table 1). Among the 19 patients, 10 had at least one previous transplant. Median age at time of last transplant was 37 years (range 27–59). In the cohort, 72% were female (*n* = 14), 78% Caucasian (*n* = 15), with 61% had live donor transplant (*n* = 12) as the last transplant. Diagnosis of aHUS was attributed to previous failure of at least one kidney transplant in 68% (*n* = 13) of patients. The clinical characteristics of the study population at the time of most recent transplant are outlined in Table 1. Median duration of follow up was 46 (range 6–237) months.

**Table 1:** Clinical characteristics of patients with and without eculizumab prophylaxis at the time of most recent transplant

Variable	Eculizumab prophylaxis (10 patients, 10 transplants)	No eculizumab prophylaxis (9 patients, 9 transplants)	*p*-value
Age, median years (range)	34 (27–50)	38 (11–59)	0.44
Female, n	8 (80%)	7 (78%)	0.91
History of prior transplant, n	6 (60%)	4 (44%)	0.50
Total number of prior transplants, n	9	7	–
Caucasian, n	10 (100%)	5 (56%)	**0.01**
African-American, n	0 (0%)	4 (44%)	
Cause of ESRD, n			
aHUS	8 (80%)	5 (56%)	0.57
HUS/TTP	1 (10%)	2 (22%)	
FSGS	1 (10%)	1 (11%)	
HTN	0 (0%)	1 (11%)	
Complement mutations, n			
CFH	4 (40%)	3 (33%)	0.25
CFHR3-CFHR1	2 (20%)	0 (0%)	
MCP	0 (0%)	1 (11%)	
THBD	0 (0%)	1 (11%)	
No mutation detected	4 (40%)	4 (44%)	
Induction therapy, n			
Thymoglobulin	9 (90%)	6 (66%)	0.18
Basiliximab	1 (10%)	0 (0%)	
Daclizumab	0 (0%)	2 (22%)	
Maintenance therapy, n			
Eculizumab, Tacrolimus, MMF, prednisone	10 (100%)	0 (0%)	–
Tacrolimus, MMF, prednisone	0 (0%)	7 (78%)	
Cyclosporine, MMF, prednisone	0 (0%)	1 (11%)	
Rapamycin, MMF, prednisone	0 (0%)	1 (11%)	
Duration of eculizumab, n			
6 months	3 (30%)	–	–
6 to 30 months	1 (10%)	–	
Lifelong	6 (60%)	–	
Plasmapheresis, n	0 (0%)	5 (56%)	**0.006**

*aHUS* atypical haemolytic uremic syndrome, *HUS/TTP* haemolytic uremic syndrome/thrombotic thrombocytopenia purpura, *FSGS* focal segmental glomerulosclerosis, *HTN* hypertension, *MMF* Mycophenolate mofetil

Elven patients had genetic complement mutations, 37% (*n* = 7) were noted to have complement factor H (CFH) mutations, 21% (*n* = 4) had other complement factor mutations or CFH related gene deletions and 42% (*n* = 8) had no mutations identified on genetic testing (CFH, CFHR3-CFHR1, MCP and THBD).

Since the FDA approval of eculizumab in the aHUS treatment, all patients with aHUS as the cause of ESRD who underwent kidney transplantation in our center received eculizumab prophylactic therapy. The only exception was one patient with membrane cofactor protein (MCP or CD46) mutation. A total of 56% (10 of 19 patients) at the time of most recent transplant received prophylactic eculizumab. Eculizumab was discontinued in 3 patients at 6 months post-transplant, where no genetic mutation was identified, while the other 7 patients remained on lifelong eculizumab prophylactic therapy due to identified complement mutations. Median follow up in this group was 39.5 (range 4–88) months, with no aHUS recurrence during the follow up. Mean estimated glomerular filtration rate (eGFR) at the time of last follow up was 59.5 ml/min/m^2^ (Table 2). Only, one patient developed ESRD after developing T cell mediated rejection and did not have evidence of TMA on biopsy or other signs of aHUS recurrence. Nine patients did not receive eculizumab prophylaxis in the most recent transplant. The outcomes of the most recent transplant compared to those of the eculizumab group presented in Table 2.

**Table 2:** Outcomes of most recent transplant in patients treated with eculizumab prophylaxis pre-transplant versus patients not treated with eculizumab prophylaxis pre-transplant

Variable	Eculizumab prophylaxis (10 patients)	No eculizumab prophylaxis (9 patients)	*p*-value
Follow-up post-transplant, median years (range)	3.48 (0.36–7.21)	3.80 (1.30–14.70)	0.33
aHUS recurrence	0 (0%)	2 (22%)	0.15
Graft outcome			
Median creatinine, mg/dL (range)	1.3 (0.9–2.0)	1.1 (0.7 to 2.1)	0.46
Median eGFR, ml/min/m2 (range)	55 (43–76)	61 (32–92)	0.55
Hemodialysis, (n)	1 (10%)	4 (44%)	0.09
Death, (n)	0 (0%)	0 (0%)	–

*aHUS* atypical haemolytic uremic syndrome, *eGFR* estimated glomerular filtration rate

We compared the recurrence of aHUS across all allograft incidents, including prior allografts and the most recent allograft incident in all patients (total of 24 allograft incidents without eculizumab prophylaxis and 10 allograft incidents with eculizumab prophylaxis). Recurrent aHUS occurred in 17 allograft (in 13 patients) out of total of 24 (70%) allograft incidents without eculizumab prophylaxis; no recurrence occurred in the 10 allografts incidents treated with eculizumab prophylaxis (*p* < 0.001). In the non-prophylactic group, only 3 out of these allograft incidents were treated with eculizumab at the time of biopsy proven identification of aHUS recurrence post-transplant. Out of these 3 patients, only one did not respond to therapy, as eculizumab was utilized very late in the course, and progressed to ESRD. The second patient responded very well to eculizumab treatment but allograft failed later due to recurrent kidney allograft insults. The third patient responded very well to eculizumab treatment and allograft function remained excellent. Prior to eculizumab era, 14 incidents were treated with plasmapheresis (no renal recovery in any of these patients).

**Fig. 1:**
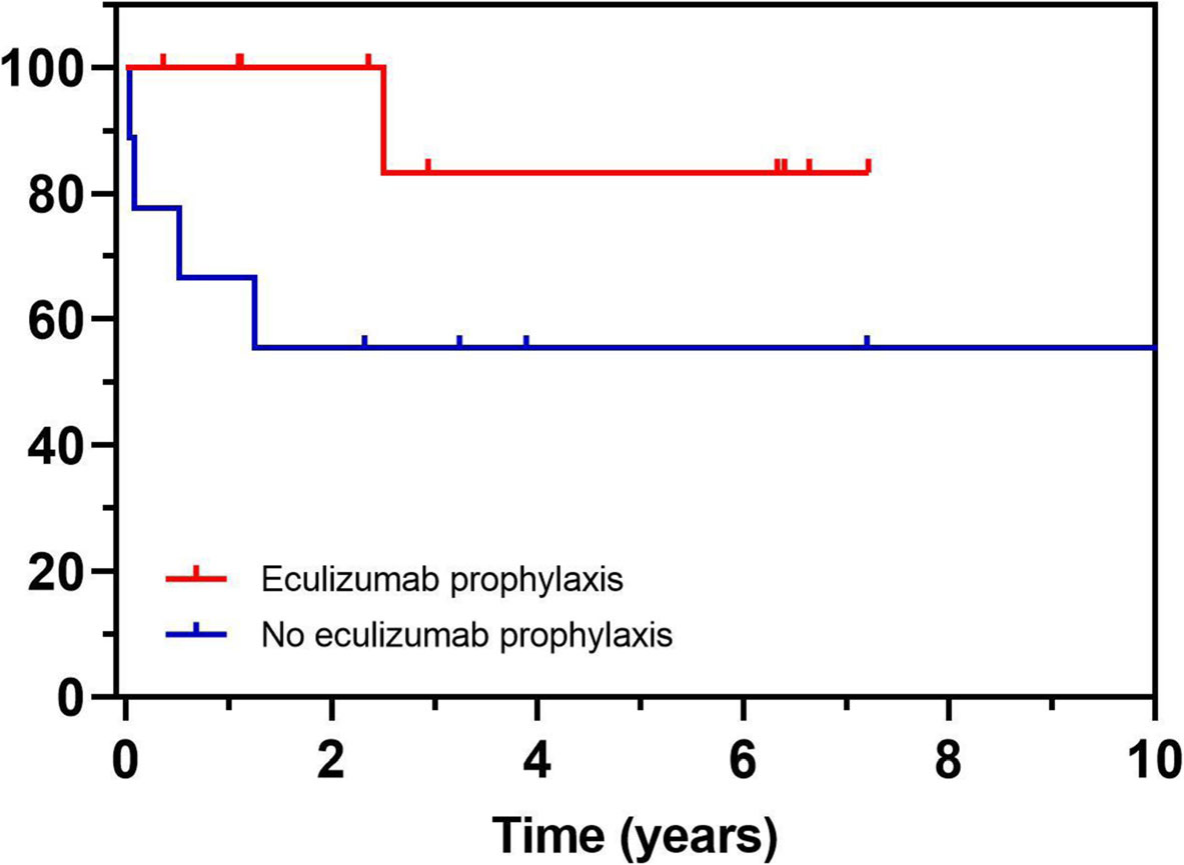
Kaplan-Meier curve demonstrating graft survival after most recent transplant in patients with and without eculizumab prophylaxis. Graft failure occurred in 4 of 9 patients without eculizumab prophylaxis and 1 of 10 patients with eculizumab prophylaxis over the follow up period (*p* = 0.09)

At the end of the follow up period, only 3 allografts were deemed functional in the non-eculizumab prophylactic group, in contrast, other than one allograft failure in the prophylactic group, all 9 allografts are still functioning, Fig. 1.

Median duration of eculizumab therapy in both groups was 13 (range 1–76) months. At the end of study period, 60% (*n* = 6) of patients who received Eculizumab continue to be on treatment, this treatment is considered lifelong in all these cases until more data is available.

Over the follow up period since the most recent transplant, there were no serious infections related to eculizumab treatment was observed. However, one patient had varicella zoster virus treated as an outpatient. There were no incidents of infections secondary to encapsulated organisms as a result of eculizumab treatment.

## Discussion

Eculizumab is highly effective in treating patients with aHUS [4, 7, 11]. Its use in post-transplant recurrence of disease was first demonstrated a decade ago [6]. Subsequently, multiple case reports and case series have demonstrated the use of Eculizumab in this patient population [6, 12, 13].

We herein report a long term, single center experience with Eculizumab in prevention of aHUS in kidney transplant recipients with close to 4 years of median follow up and individual duration of treatment extending beyond 7 years. We demonstrated effective prevention of aHUS without increase in infectious complications.

In accordance with recent observational data from the Global aHUS registry, the group of patients that underwent prophylactic Eculizumab therapy (denoted as group 1 in the registry) had better outcomes [14]. None of the patients in our cohort had recurrence of aHUS in the post-transplant period or were commenced on dialysis during the follow up period. Additionally, mean eGFR at the time of last follow up was 59.5 ml/min/m^2^, which was very similar to group 1 is the Global aHUS registry study (mean eGFR 60.6 ml/min/m^2^ at 6 months).

In spite of the two allograft failures in the two patients who received eculizumab for post-trasplant aHUS recurrence in our cohort, eculizumab remained the best most effective therapy for recurret aHUS. Similar findings were established in the Global aHUS registry, in this study 344 received one or more kidney transplant,188 had received eculizumab. In this study, 88 patients (47%) were received eculizumab before and during their most recent transplant (group 1). On the other hand 100 patients (53%; group 2) were treated with eculizumab post-transplantation for recurrent aHUS. Alloraft function within 6 months of transplantation was significantly better in group 1 compared with group 2, and allograft survival was significantly better. One meningococcal infection and 3 deaths (unrelated to eculizumab) were reported [14]. This further substantiates the evidence that eculizumab prophylaxis prevented aHUS recurrence and led to better graft outcomes.

Outcomes in patients treated with plasma exchange (PLEX) alone in the post-transplant period were especially poor, with none of the patients achieving renal recovery. PLEX was not efficacious in a French study of 146 patients with aHUS, with 44% of patients receiving PLEX developed ESRD at the first episode of aHUS [15]. In another study, irrespective of PLEX being used, half of patients with post-transplant thrombotic microangiopathy had allograft failure within a year [2].

More than a third of patients in our cohort had complement factor H (CFH) mutations, accounting for a majority of the identified mutations. Previous studies have shown that CFH is the most prevalent mutation leading to development of aHUS [16–18]. Additionally, studies have revealed that the highest graft failure rates have been in patients with CFH mutations- 71% failure at 1 year [19].

In our study, out of the 10 patients treated with eculizumab prophylactically and continued treatment post-transplantation, therapy was ceased in 3 patients within 6–12 months of engraftment, given no mutation detected. In the subsequent follow up period, none of the patients developed recurrence of disease. In a previous case series, a patient with no complement mutation has cessation of prophylactic eculizumab after 28.7 months, with no recurrence during 9 months of follow up [20]. With the longest demonstrated follow up to our knowledge (median 40 months), our study shows that cessation of prophylaxis may be possible with minimal risk of recurrence, in carefully selected patients with no identified complement mutations.

None of the patients treated with Eculizumab in our study had episodes of meningococcal infection. It is being increasingly recognized that meningococcal infection appears to be an uncommon event in patients treated with Eculizumab, whether it be for aHUS or paroxysmal nocturnal hemoglobinuria [3, 4, 7, 11, 21]. Nevertheless, vigilance for this infection should be maintained since, as demonstrated in previous studies, the risk cannot be deemed non-existent [14, 22].

The limitations of this study include the small sample size, retrospective and observational nature of the study and limited to practice at one center, which might have changed over the long period of the study.

## Conclusions

In conclusion, this study demonstrates one of the longest follow up of kidney transplant recipients treated with Eculizumab reported till date. This further consolidates the safety and efficacy of prophylactic therapy to prevent aHUS recurrence in this patient population. Additionally, it provides a direction for possible cessation of therapy in carefully selected patients with no evidence of complement mutations.

## Data Availability

All data generated or analysed during this study are included in this published article.

## Abbreviations

aHUS: Atypical hemolytic uremic syndrome
ESRD: End stage renal disease
KTR: Kidney transplant recipient
IRB: Institutional Review Board
TMA: Thrombotic microangiopathy
IVIg: Intravascular immunoglobulin
CFH: Complement factor H
eGFR: Estimated glomerular filtration rate
PLEX: Plasma exchange
